# Stromal Fibroblasts from the Interface Zone of Triple Negative Breast Carcinomas Induced Epithelial-Mesenchymal Transition and its Inhibition by Emodin

**DOI:** 10.1371/journal.pone.0164661

**Published:** 2017-01-06

**Authors:** Hsiang-Chi Hsu, Liang-Chih Liu, Hao-Yu Wang, Chao-Ming Hung, Ying-Chao Lin, Chi-Tang Ho, Tzong-Der Way

**Affiliations:** 1 Department of Biological Science and Technology, College of Biopharmaceutical and Food Sciences, China Medical University, Taichung, Taiwan; 2 Department of Surgery, China Medical University Hospital, Taichung, Taiwan; 3 Department of Medicine, College of Medicine, China Medical University, Taichung, Taiwan; 4 Department of General Surgery, E-Da Hospital, I-Shou University, Kaohsiung, Taiwan; 5 School of Medicine, I-Shou University, Kaohsiung, Taiwan; 6 Division of Neurosurgery, Buddhist Tzu Chi General Hospital, Taichung Branch, Taiwan; 7 School of Medicine, Tzu Chi University, Hualien, Taiwan; 8 Department of Medical Imaging and Radiological Science, Central Taiwan University of Science and Technology, Taichung, Taiwan; 9 Department of Food Science, Rutgers University, New Brunswick, New Jersey, United States of America; 10 Department of Health and Nutrition Biotechnology, Asia University, Taichung, Taiwan; National Cheng Kung University, TAIWAN

## Abstract

“Triple negative breast cancer” (TNBC) is associated with a higher rate and earlier time of recurrence and worse prognosis after recurrence. In this study, we aimed to examine the crosstalk between fibroblasts and TNBC cells. The fibroblasts were isolated from TNBC patients’ tissue in tumor burden zones, distal normal zones and interface zones. The fibroblasts were indicated as cancer-associated fibroblasts (CAFs), normal zone fibroblasts (NFs) and interface zone fibroblasts (INFs). Our study found that INFs grew significantly faster than NFs and CAFs *in vitro*. The epithelial BT20 cells cultured with the conditioned medium of INFs (INFs-CM) and CAFs (CAFs-CM) showed more spindle-like shape and cell scattering than cultured with the conditioned medium of NFs (NFs-CM). These results indicated that factors secreted by INFs-CM or CAFs-CM could induce the epithelial-mesenchymal transition (EMT) phenotype in BT20 cells. Using an *in vitro* co-culture model, INFs or CAFs induced EMT and promoted cancer cell migration in BT20 cells. Interestingly, we found that emodin inhibited INFs-CM or CAFs-CM-induced EMT programming and phenotype in BT20 cells. Previous studies reported that CAFs and INFs-secreted TGF-β promoted human breast cancer cell proliferation, here; our results indicated that TGF-β initiated EMT in BT20 cells. Pretreatment with emodin significantly suppressed the TGF-β-induced EMT and cell migration in BT20 cells. These results suggest that emodin may be used as a novel agent for the treatment of TNBC.

## Introduction

Breast cancer is the most commonly diagnosed cancer in women and affects the lives of millions of women worldwide each year. The specific group accounts for approximately 15–20% of all breast cancer is triple-negative breast cancer (TNBC). TNBC is defined by the lack of demonstrable expression of the estrogen receptor (ER), progesterone receptor (PR) or HER2 proteins [1]. TNBC has a higher rate of distant recurrence and shorter overall survival in the metastatic setting compared with other subtypes of breast cancer. Metastatic TNBC is an aggressive disease and the median survival is less than one year. Almost all TNBC patients die from the progression of their disease despite adjuvant chemotherapy [2]. Therefore, novel anti-cancer drugs with higher efficiency and specificity are urgently needed.

Recent studies indicated that solid tumors comprised not only neoplastic cells but also surrounded by a variety of non-neoplastic cells, most notably fibroblasts, adipocytes, endothelial cells, pericytes, mesenchymal stem cells (MSCs) and immune cells that constitute a ‘tumor microenvironment’. The crosstalk between neoplastic cells and non-neoplastic cells plays an important role in tumor progression, and responses to antitumor therapy [3, 4]. The fibroblast is one of the most crucial components of tumor microenvironment, which promotes the remodeling of extracellular matrix (ECM) and produces paracrine growth factors that control cell proliferation, survival and death [5]. Such fibroblasts, known as cancer-associated fibroblasts (CAFs), have been reported to be associated with the progression of various cancer types such as prostate [6–8], pancreatic [9], head and neck [10] and breast cancers [11]. These results suggest that the stromal fibroblasts in tumor tissues possess biological characteristics distinct from those of normal fibroblasts. However, the specific functional contributions of fibroblasts located in the interface zone between the normal zone and the tumor invasion front remain largely unknown.

An approximately 90% of breast cancer deaths are caused by local invasion and distant metastasis, however, the mechanism underlying this event remains poorly defined. Epithelial-mesenchymal transition (EMT), a cellular process critical to normal morphogenesis, was recognized as an important mechanism for the initial step of metastasis [12, 13]. EMT results in loss of features characteristic of epithelial cells and acquisition of a mesenchymal nature. Recent studies have examined EMT in tumor invasion, chemoresistance, and the relationship between cancer stem cells [14–16]. Some signals received from tumor microenvironments, such as tumor necrosis factor α (TNFα), transforming growth factor β (TGFβ), IL-6, fibroblast growth factor (FGF) and epidermal growth factor (EGF), can trigger EMT [17–19]. It is important to examine the signals mediated by these microenvironment stimuli in initiating and controlling EMT and cancer metastasis.

Emodin (1,3,8-trihydroxy-6-methylanthraquinone) is an anthraquinone derivative present in the root and rhizome of *Rheum palmatum* L. (Polygonaceae). This herb is widely used in traditional Chinese and Japanese medicine. Emodin possesses a number of biological activities such as antiviral, anti-inflammatory, anti-ulcerogenic, immunosuppressive, pro-apoptotic and chemopreventive activities [20]. However, so far there is little evidence showing the possible effects of emodin on tumor invasion and metastasis.

In the present study, we tested whether fibroblasts isolated from TNBC patients’ tissues in tumor burden zones (CAFs), distal normal zones (NFs) and interface zones (INFs) contributed distinctive microenvironmental influences on TNBC. Our results demonstrated that fibroblasts isolated from different zones differed with respect to their ability to induce EMT. Moreover, we also tested whether emodin could inhibit the ability of different fibroblasts promoting TNBC progression. Our results found that emodin inhibited EMT induced by CAFs or INFs. These findings suggest that emodin is a promising candidate for TNBC prevention.

## Materials and Methods

### Reagents and antibodies

The compounds emodin and 4’,6-diamidino-2-phenylindole (DAPI) were purchased from Sigma Chemical Co. (St. Louis, MO, USA). Human recombinant TGF-β was purchased from R&D Systems (Minneapolis, MN, USA). Primary antibodies against Snail, E-cadherin, β-cadherin, MMP-2 and Slug were purchased from Cell Signaling Technology (Beverly, MA, USA). Primary antibody against vimentin was purchased from Abcam Inc. (Cambridge, MA, USA). Primary antibodies against Twist were purchased from Santa Cruz Biotechnology (Santa Cruz, CA, USA). Primary antibody against β-actin was purchased from Sigma Chemical Co. (St. Louis, MO, USA). Secondary antibodies, HRP-conjugated Goat anti-Mouse IgG and Goat anti-Rabbit IgG, were obtained from Millipore (Billerica, MA, USA).

### Cell lines and cell cultures

BT20 cells (ATCC® HTB-19™) were purchased from American Type Culture Collection (Manassas, VA, USA). BT20 cells were cultured in DMEM/F12 supplemented with 10% fetal bovine serum (FBS). Cells were grown in a humidified incubator which provided an atmosphere of 5% CO_2_ at a constant temperature of 37°C. Materials used for cell cultures were purchased from Invitrogen (Burlington, Ontario, Canada).

### Isolation of primary fibroblasts

TNBC patients’ tissues were obtained from patients undergoing surgery at China Medical University Hospital, Taiwan. The protocol for the study was approved by the Institutional Review Board (IRB) of China Medical University Hospital (CMUH-104-REC2–121). All participants signed written informed consent forms detailing tissue use for comprehensive experiments on breast cancer. We obtained tumor specimens from three zones: the normal zone (at least 10 mm distal normal tissue from the outer tumor boundary), interface zone (adjacent tissue within 5 mm of the outer tumor boundary) and tumor zone (tissue within the tumor boundary). The tumor specimens were fixed in formalin and embedded in paraffin for routine histopathological analysis. The remnant was used to isolate primary fibroblasts as described previously [21].

### Confocal microscopy

Cells were fixed for 20 min in 3% formaldehyde in PBS, permeabilized in 0.2% Triton X-100/PBS for 5 min, and blocked with 3% FBS for 20 minutes. The expression of E-cadherin or vimentin in the cells was analyzed through Leica confocal microscopy conducted using a monoclonal primary antibody. Nuclear staining was done with DAPI.

### Direct co-culture of fibroblasts and BT20 cells

The CAFs, NFs, and INFs were directly co-cultured with BT20 cells as described previously [21]. CAFs, NFs, INFs or BT20 cells were incubated with serum-free DMEM/F12 containing 5 μM CellTracker Green CMFDA (5-chloromethylfluorescein diacetate; Invitrogen, Burlington, Ontario, Canada) for 45 minutes at 37°C. The solution was replaced with fresh, prewarmed medium for an additional 2 h. The cells were washed twice with PBS and then unstained fibroblasts or BT20 cells were seeded onto plates containing CMFDA-stained fibroblasts or BT20 cells, respectively. Finally, the co-cultures were incubated with medium (DMEM/F12, 1% FBS and 100 IU/mL penicillin with 100 μg/mL streptomycin) for 1 week. Using confocal microscopy, CMFDA-stained cells were easily distinguished from unstained cells.

### Wound healing assay

BT20 cells grown to approximately 50% confluence were stained with CMFDA and then co-cultured with CAFs, NFs, or INFs, respectively. Cells were allowed to grow to 100% confluence and then scratched by a sterile pipette tip and rinsed with PBS to remove cellular debris. Allow cells to grow and close the wound for 24 h. Wound closure was measured in ten random fields at 200X magnification using Image-Pro Express software and a NIKON TE2000-U Inverted Microscope. Data of three independent experiments were analyzed by *t*-test using GraphPad Prism 5 software.

### Western blot analysis

Briefly, cells in 10-cm culture dishes (1 × 10^6^ per dish) were treated with the indicated in figure legends. Fifty micrograms of protein extract was loaded into sodium dodecyl sulphate-polyacrylamide gels, and the separated proteins were transferred to nitrocellulose filters. The filters were probed with the appropriate primary antibody. Western blotting was conducted as recently described [22].

### Statistical analysis

All values were expressed as mean ±S.D. Each value was the mean of at least three individual experiments in each group. Student’s *t*-test was used for statistical comparison. Asterisk indicates that the values were significantly different from the control (*, *P* < 0.05; **, *P* < 0.01; ***, *P* < 0.001).

## Results

### Comparison of primary fibroblasts isolated from different zones of TNBC patients’ tissues

**Fig 1:**
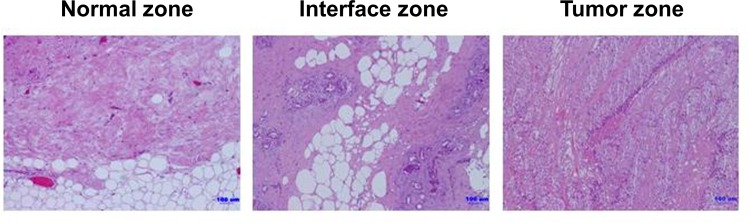
Collection of representative TNBC patient’s tissue. TNBC patient’s tissue was grossly divided into three distinct regions: the tumor zone, interface zone and normal zone. For subsequent fibroblasts isolation, the histological analysis was confirmed by hematoxylin and eosin (H&E) staining and a representative sample of tissue was collected from three distinct regions.

**Fig 2:**
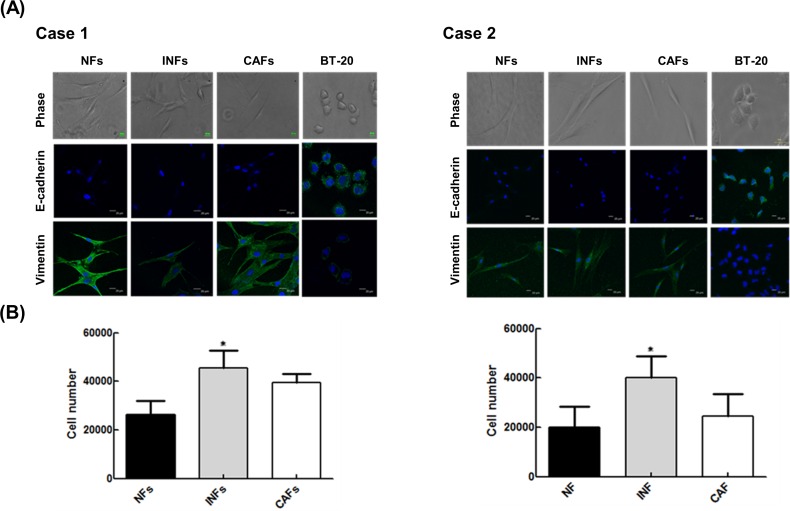
Comparison of primary fibroblasts from three distinct regions. (A) Normal zone fibroblasts (NFs), interface zone fibroblasts (INFs) and cancer-associated fibroblasts (CAFs) were obtained from two patients. Each type of fibroblasts and BT20 cells were examined by phase-contrast microscopy and immunostaining for E-cadherin and vimentin; nuclei were stained with DAPI. (B) NFs, INFs, and CAFs seeded at the same density (5000 cells/well) in 6-well plates for 5 days. Cell numbers were calculated by Trypan Blue assay under the same experimental conditions. The number of INFs was increased relative to NFs and CAFs (*, *p* < 0.05).

We first obtained representative TNBC patients’ tissues from three zones: the tumor zone, interface zone and normal zone (Fig 1). Fibroblasts were isolated from TNBC patients’ tissues in the normal, interface and tumor zones, which were correspondingly designated as NFs, INFs and CAFs, respectively. Our study found that each type of fibroblast obtained from two patients possessed the basic fibroblast characteristics of an identical and long spindle-shaped morphology. Moreover, the strong expression of fibroblastic marker vimentin and being negative for epithelial marker E-cadherin in each type of fibroblast (Fig 2A). Oppositely, a human mammary epithelial cell line BT20 cells displayed an up-regulation of epithelial marker (Fig 2A). To compare the growth rate of primary fibroblasts isolated from different zones, we counted the cell number. After seeding at the same density (5,000 cells/well) in 6-well plates and culturing for 4 days, the number of INFs was increased relative to NFs and CAFs (Fig 2B).

### INFs or CAFs induced EMT programming and phenotype in TNBC cells

**Fig 3:**
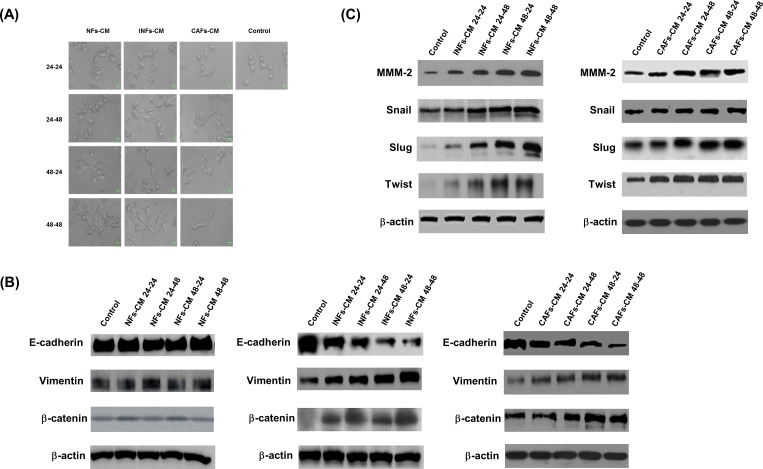
INFs and CAFs induced EMT programming and phenotype in BT20 cells. (A) The conditioned medium of NFs (NFs-CM), INFs (INFs-CM) and CAFs (CAFs-CM) were collected in 24 h and 48 h and used to culture BT20 cells for 24 h (24–24, 48–24) and 48 h (24–48, 48–48). Phase-contrast pictures were taken using a 10^3^ objective. (B) BT20 cells were treated with NFs-CM, INFs-CM and CAFs-CM for indicated time periods. The cells were then harvested and lysed for the detection of E-cadherin, vimentin, β-catenin, and β-actin. (C) BT20 cells were treated with NFs-CM, INFs-CM and CAFs-CM for indicated time periods. The cells were then harvested and lysed for the detection of MMP-2, snail, slug, twist, and β-actin. Western blot data presented are representative of those obtained in at least 3 separate experiments.

Recent study found that CAFs promoted aggressive phenotypes of breast cancer cells through EMT [23]. To determine whether NFs, INFs and CAFs had different capacities to modulate tumor progression, the conditioned medium of NFs (NFs-CM), INFs (INFs-CM) and CAFs (CAFs-CM) were collected and used to culture BT20 cells. As shown in Fig 3A and S1 File, the epithelial BT20 cells cultured with INFs-CM or CAFs-CM showed more spindle-like shape and cell scattering than cultured with NFs-CM. To investigate the changes of EMT phenotype induced by NFs-CM, INFs-CM and CAFs-CM in BT20 cells, we examined the expression of epithelial marker E-cadherin, mesenchymal marker vimentin and β-cadherin. Our results showed that BT20 cells cultured with INFs-CM or CAFs-CM had decreased expression of epithelial marker E-cadherin, and increased expression of mesenchymal marker vimentin and β-cadherin (Fig 3B). The expression levels of mesenchymal marker MMP2, Snail, Slug and Twist were upregulated in BT20 cells cultured with INFs-CM or CAFs-CM (Fig 3C). These factors secreted by INFs-CM or CAFs-CM could induce the EMT phenotype in BT20 cells.

### INFs or CAFs induced EMT programming and phenotype in BT20 cells in an *in vitro* co-culture model

**Fig 4:**
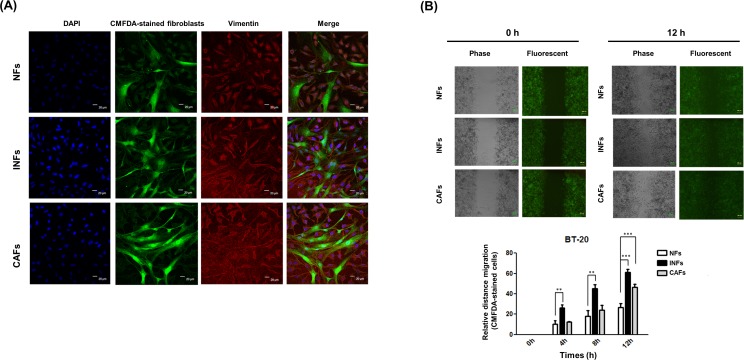
INFs and CAFs induced EMT programming and phenotype in an *in vitro* co-culture model. (A) The CAFs, NFs, and INFs were directly co-cultured with BT20 cells. The adherent CAFs, NFs, and INFs or BT20 cells were stained by 5 μM CMFDA. CMFDA-stained fibroblasts (green) were distinguished from unstained cancer cells. Immunofluorescence staining for vimentin (red) in co-cultures of BT-20 and fibroblasts. (B) BT20 cells grown to approximately 50% confluence were pre-stained with CMFDA and then co-cultured with CAFs, NFs, or INFs. Cells were allowed to grow to 100% confluence and then scratched by a plastic tip and washed by PBS to remove cell debris. The cells were incubated for 12 h to allow cells to grow and close the wound. The cell motility data are plotted as means with S.D.

To further determine whether NFs, INFs and CAFs had different capacities to induce the EMT phenotype, we co-cultured NFs, INFs and CAFs directly with BT20 cells, allowing direct cancer cells and fibroblasts interaction. We examined the expression of mesenchymal marker vimentin (red) after co-culture BT20 cells and CMFDA-stained (green) fluorescent fibroblasts by immunofluorescence staining. Direct co-culture of BT20 cells with INFs or CAFs showed higher percentage of vimentin-positive cells than did those co-cultured with NFs (Fig 4A). The results indicated that INFs or CAFs induced EMT programming and phenotype in co-cultured BT20 cells.

### INFs or CAFs promoted cancer cell migration under co-culture conditions

Cancer cells enhanced ability of migration and invasion through EMT [12]. To examine whether NFs, INFs and CAFs promoted BT20 cells migration, we performed a wound healing assay under direct co-culture of BT20 cells with different types of fibroblasts. After scratching, BT20 cells were allowed to recover and their capacity to migrate and fill the wound area was assessed. Direct co-culture of BT20 cells with INFs or CAFs, phase contrast microscopic observation indicated that the size of the wound area was lower than those co-cultured with NFs (Fig 4B). Moreover, fluorescent photomicrographs showed that CMFDA-stained BT20 cells (green) were observed to migrate into the wound area in the co-cultures of INFs or CAFs (Fig 4B). The results indicated that INFs and CAFs were able to promote BT20 cell migration. Interestingly, INFs possessed the more potent effect on migratory behavior than CAFs.

### EMT programming and phenotype were blocked by emodin in BT20 cells cultured with INFs-CM or CAFs-CM

**Fig 5:**
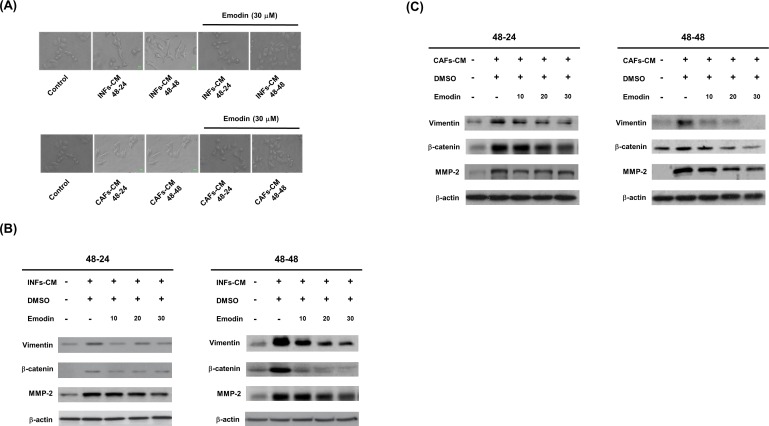
Emodin inhibited INFs and CAFs induced EMT programming and phenotype in BT20 cells. (A) The changes in cell morphology were assessed following treatment of BT20 cells with INFs-CM or CAFs-CM for indicated time periods with or without 30 μM emodin. Emodin can reverse the EMT properties from fibroblastic shape recover to the cobble-stone shaped. (B) BT20 cells were treated with INFs-CM for indicated time periods with or without 30 μM emodin. (C) BT20 cells were treated with CNFs-CM for indicated time periods with or without 30 μM emodin. The cells were then harvested and lysed for the detection of vimentin, β-catenin, MMP-2, and β-actin. Western blot data presented are representative of those obtained in at least 3 separate experiments.

Previous study indicated that emodin inhibits the migration, invasion and metastasis of TNBC cells [24]. To test whether INFs-CM or CAFs-CM-induced EMT programming and phenotype were blocked by emodin. Emodin was added to INFs-CM or CAFs-CM for culturing BT20 cells. Then, the changes in cell morphology were assessed following treatment of BT20 cells with INFs-CM or CAFs-CM with or without emodin for 24 and 48 h. Emodin inhibited INFs-CM or CAFs-CM-induced BT20 cells spindle-like shape and cell scattering (Fig 5A). We next examined the expression of mesenchymal markers (vimentin, β-catenin and MMP-2) and found that increased mesenchymal markers stimulated by INFs-CM were reversed by adding emodin in BT20 cells in a dose and time-dependent manner (Fig 5B). Furthermore, increased mesenchymal markers stimulated by CAFs-CM were reversed by adding emodin in BT20 cells in a dose and time-dependent manner (Fig 5C). These results indicated that emodin inhibited INFs-CM or CAFs-CM-induced EMT programming and phenotype in BT20 cells.

### TGF-β induced EMT programming and phenotype in BT20 cells

**Fig 6:**
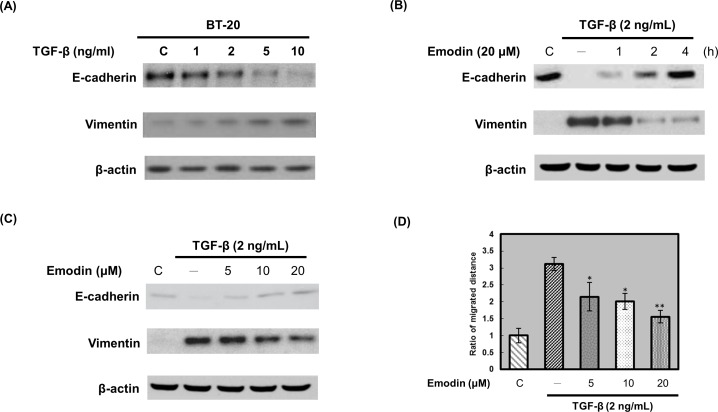
Emodin blocks TGF-β-induced EMT in BT20 cells. (A) BT20 cells were treated with various concentrations (1–10 ng/mL) of TGF-β for 24 h. The cells were then harvested and lysed for the detection of E-cadherin, vimentin, and β-actin. (B) BT20 cells were pretreated with DMSO (control) or 20 μM emodin for indicated time periods and then stimulated with TGF-β (2 ng/mL) for 24 h. The cells were harvested and lysed for the detection of E-cadherin, vimentin, and β-actin. (C) BT20 cells were pretreated with DMSO (control) or increasing emodin concentrations (5–20 μM) for 2 h and then stimulated with TGF-β (2 ng/mL) for 24 h. The cells were harvested and lysed for the detection of E-cadherin, vimentin, and β-actin. Western blot data presented are representative of those obtained in at least 3 separate experiments. (D) For wound healing assay, confluent BT20 monolayer was pretreated with DMSO (control) or increasing emodin concentrations (5–20 μM) for 2 h, cells were scratched by pipette tips and washed to remove the debris and following by fresh medium containing 0.5% serum with emodin. Cells were then incubated with 2 ng/mL TGF-β for 24 h. TGF-β-induced cell motility was determined by measuring the closure of wound. Data were plotted by mean ± S.D. (*n* = 3). The closure distance of the control cells was set to 1. Emodin significantly inhibited TGF-β-induced cell motility (*, *p* < 0.05; **, *p* < 0.01).

CAFs and INFs-secreted TGF-β promote human breast cancer cells proliferation [25–27]. We next determined whether CAFs and INFs-secreted TGF-β initiated EMT in TNBC cells. The cell morphology was assessed following treatment of BT20 cells with various concentrations (1–10 ng/mL) of TGF-β for 24 h. After treatment with TGF-β, BT20 cells altered their morphology to assume more of a fibroblast-like appearance and reduced their cell-cell contact (S2 Fig in S1 File). After treatment of BT20 cells with various concentrations (1–10 ng/mL) of TGF-β for 24 h, the expressions of the epithelial phenotype marker (E-cadherin) and mesenchymal phenotype marker (vimentin) were determined. TGF-β decreased E-cadherin expression and increased vimentin expression in a dose-dependent manner (Fig 6A). These results indicated that TGF-β induced EMT programming and phenotype in BT20 cells.

### Emodin blocked TGF-β-induced EMT programming and phenotype in BT20 cells

To determine whether emodin affected the TGF-β-induced EMT programming and phenotype, BT20 cells were pretreated with emodin prior to stimulation with TGF-β. Pretreatment with emodin significantly restored the TGF-β-induced downregulation of E-cadherin and upregulation of vimentin in a dose and time-dependent manner (Fig 6B and 6C). We next examined whether emodin blocked TGF-β-induced cell migration. Pretreated confluent BT20 cells with various concentrations of emodin for 2 h, cells were scratched and then incubated with 2 ng/mL of TGF-β for 24 h. BT20 cells were allowed to recover and their capacity to migrate and fill the wound area was determined by measuring the wound closure assessed. Our results indicated that TGF-β significantly induced cell migration, however, the TGF-β-induced cell migration was inhibited by emodin in a dose-dependent manner (Fig 6D).

## Discussion

Traditionally, most anticancer drugs are designed to kill tumor cells. However, recent studies have demonstrated that cancers not only contain tumor cells but also have very complex substances with multiple components involved in tumor growth, invasion, and metastasis. Fibroblasts are a major component of solid tumors and associated with cancer cells at all stages of cancer progression. In the present study, we demonstrated the differential interactions between fibroblasts from different tumor zones and TNBC cells. Our study found that CAFs and INFs grown with human TNBC BT20 cells dramatically promoted cell migration and induced an EMT process. Under the same experimental conditions, this effect was not detected or was weaker when NFs were grown with BT20 cells. Importantly, INFs were more competent in promoting these changes in BT20 cells than were CAFs. Targeting these cells through suppressing their supportive procarcinogenic paracrine effects is mandatory for improving the current therapies that are mainly targeting TNBC. To this end, we tested the effect of emodin in suppressing the carcinogenic effects of active CAFs and INFs. We have shown that emodin inhibited INFs-CM or CAFs-CM-induced EMT programming and phenotype.

Fibroblasts are a key determinant in the malignant progression of cancer cells through the involvement in progressive genetic instability, angiogenesis [28], EMT [29], deregulation of anti-tumor immune responses [30], and remodeling of the extracellular matrix [31]. Recent reports indicated that CAFs could promote aggressive phenotypes of breast cancer cells. Yu et al demonstrated that CAFs promoted aggressive phenotypes of breast cancer cells through EMT induced by paracrine TGF-β1 [32]. Al-Ansari et al demonstrated that p16(INK4A) downregulation in breast stromal fibroblasts is an important step toward their migration and invasion by inducing EMT [33]. Lebret et al demonstrated a role for CAFs, but not for NFs, in increasing the migratory ability of PMC42-LA cells [34]. In this study, we demonstrated that CAFs enhanced the aggressive behaviors and migration in TNBC cells by inducing or promoting EMT. Collectively, these results suggested that CAFs could promote migration and invasion through induction of EMT in various types of breast cancer cells.

Compared with normal fibroblasts, our and previous data demonstrated that CAFs possess different biological properties and functions. However, the biological and molecular characterization of fibroblasts located in the interface zone remain largely unknown. In this study, we found that INFs were more potential in inducing EMT in TNBC cells than were CAFs. INFs-CM decreased the expression of E-cadherin, and increased the expression of vimentin and β-cadherin higher than that induced by CAFs-CM. Moreover, a number of studies have reported that invasion markers in tumor tissues are most dynamic and active within the interface zone, where active cancer invasion or EMT occurs [21, 35–38]. From these results, we concluded that INFs may be the primary fibroblasts involved in the remodeling of cells and tissue during invasion and metastasis of TNBC cells.

Non-toxic natural compounds that can inhibit cancer-stroma crosstalk by normalizing the tumor microenvironment may boost the traditional tumor cell–directed therapy. It has been reported that emodin significantly inhibits cell viability and induces apoptosis in several cancer cell lines [39–41]. However, so far there is little evidence on whether emodin may also influence the interaction between tumor cells and normal fibroblasts. In the current work, we clarified the role of emodin in tumor microenvironment. EMT related changes induced by INFs-CM or CAFs-CM in BT20 cells could be discerned at both morphological and molecular levels. Interestingly, emodin would reverse all INFs-CM or CAFs-CM-induced EMT related changes in BT20 cells. Chen T et al., found that emodin ameliorated glucose-induced EMT and subsequent podocyte dysfunction partly through integrin-linked kinase (ILK), which might provide a potential novel therapeutic option for diabetic kidney disease [42]. Our previous study also found that emodin inhibited TWIST1-induced EMT by inhibiting the β-catenin and Akt pathways [43]. Moreover, suppression of CK2α by the CK2α activity inhibitor emodin decreased the expression levels of vimentin and the transcription factors snail1 and smad2/3, and increased the expression of E-cadherin [44]. Taken together, these findings uncover an important role for emodin as a potent inhibitor of EMT.

Several subsequent studies established crucial roles of TGF-β-induced EMT in tumor progression. Recently, the effect of emodin on TGF-β signaling pathway and its functional relevance to proliferation, invasion and metastasis in cancer cells have been identified. Thacker PC et al., found that emodin downregulated the TGF-β activated Wnt/β-catenin signaling pathway in human cervical cancer cells [45]. Herein, in agreement with previous reports, we showed that TGF-β induced EMT in TNBC cells and this process could be effectively blocked by emodin. Emodin blocked TGF-β-induced scattering and spindle-like morphology. Moreover, TGF-β downregulated E-cadherin and upregulated vimentin was significantly inhibited by emodin.

In conclusion, it is suggested that INFs from the interface zone of the TNBC patients’ tissues may have a potential dynamic region that is a key factor leading to TNBC progression and metastasis. Emodin may prevent the activation of fibroblasts and also avert the EMT related changes induced in epithelial tumor cells by INFs-CM. These results lead us to suggest emodin as a potential new treatment agent for TNBC.

## Supporting Information

S1 File Supplementary Data. (DOCX)

## Abbreviations

CAFs: cancer-associated fibroblasts
DAPI: 4’,6-diamidino-2-phenylindole
ECM: extracellular matrix
EGF: epidermal growth factor
EMT: epithelial-mesenchymal transition
ER: estrogen receptor
FBS: fetal bovine serum
HER2: human epidermal growth factor receptor 2
INFs: interface zone fibroblasts
MTT: 3-(4,5-dimethylthiazol-2-yl)-2,5-diphenyl tetrazolium bromide
NFs: normal zone fibroblasts
PR: progesterone receptor
TGFβ: transforming growth factor β
TNBC: Triple negative breast cancer
TNFα: tumor necrosis factor α
