# Supplementary material for: Stromal Fibroblasts from the Interface Zone of Triple Negative Breast Carcinomas Induced Epithelial-Mesenchymal Transition and its Inhibition by Emodin
Source: PLoS One. 2017 Jan 6;12(1):e0164661. doi: 10.1371/journal.pone.0164661 (PMC5218416; doi:10.1371/journal.pone.0164661)
Supplement: S1 File — (DOCX) [file pone.0164661.s001.docx]

**Supporting Information**

**S1 Fig**


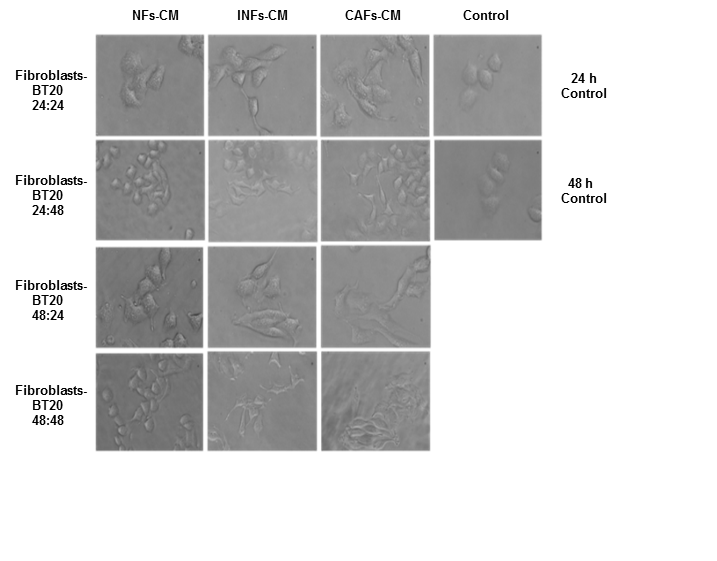


**S1 Fig. INFs and CAFs induced an EMT process in BT20 cells**. (A) The conditioned medium of NFs (NFs-CM), INFs (INFs-CM) and CAFs (CAFs-CM) were collected in 24 h and 48 h and used to culture BT20 cells for 24 h (24-24, 48-24) and 48 h (24-48, 48-48). Phase-contrast pictures were taken using a 10^3^ objective.

**S2 Fig**


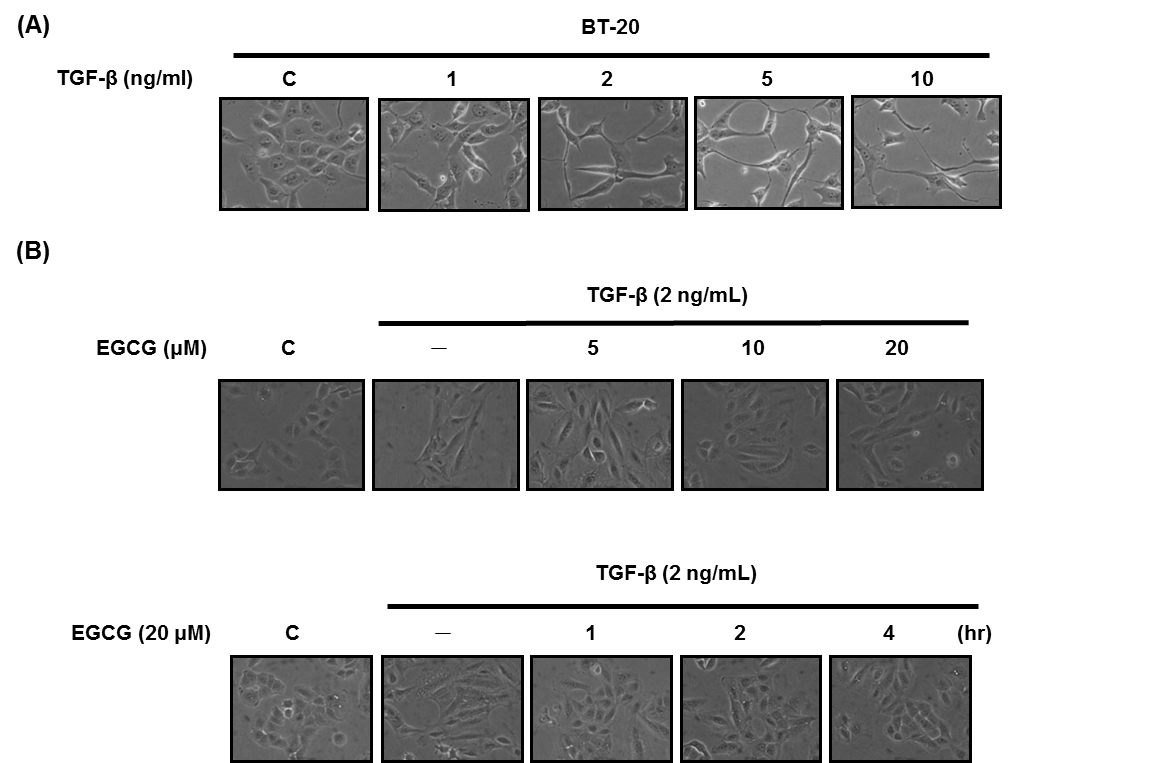


**S2 Fig.** BT20 cells undergo EMT in response to TGF-β. BT20 cells were treated with DMSO (control) as a control, or various concentrations (1–10 ng/mL) of TGF-β for 24 h. Phase-contrast pictures were taken using a 10^3^ objective.
